# Concomitant occurrence of appendiceal mucocele and ulcerative colitis: Case reports

**DOI:** 10.22088/cjim.14.2.391

**Published:** 2023

**Authors:** Hafez Fakheri, Zohreh Bari, Mohammad Yaghoobi, Parham Rabiee

**Affiliations:** 1 Gut and liver Research Center, Non-communicable Research Institute, Mazandaran University of Medical Sciences, Sari, Iran; 2 Division of Gastroenterology, Department of Medicine, McMaster University Medical Center, Hamilton, Ontario, Canada; 3 Department of Radiology, Shaheed Rajaie Cardiovascular and Medical Research Center, Tehran, Iran

**Keywords:** appendiceal, mucocele, ulcerative colitis

## Abstract

**Background:**

Appendiceal mucocele (AM) is a rare disease, manifested by accumulation of mucus in appendiceal lumen. The role of ulcerative colitis (UC) in the occurrence of appendiceal mucocele is not known. However, it is suggested that AM may be a presentation of colorectal cancer in IBD patients.

**Case Presentation:**

Here, we presented 3 cases of concomitant AM and ulcerative colitis. The first patient was a 55-year-old woman with 2-year history of left sided UC; the second person was a 52-year-old woman with 12-year history of pan-UC; and the third patient was a 60-year-old man with 11-year history of pan-colitis. They were all referred due to indolent right lower quadrant abdominal pain. Imaging evaluations suggested the presence of appendiceal mucocele and therefore, they all went under operation. Pathologic evaluation reported AM of mucinous cyst adenoma type; low-grade appendiceal mucinous neoplasm of appendix with intact serosa; and cyst-adenoma type AM for the three above-mentioned patients, respectively.

**Conclusion:**

Although concomitant occurrence of AM and ulcerative colitis is rare, regarding the potential of neoplastic changes in AM, physicians must keep in mind the diagnosis of AM in UC patients with non-specific abdominal RLQ pain or bulged appendiceal orifice during colonoscopy.

Appendiceal mucocele refers to the distended, mucus-filled appendiceal lumen, caused by either a benign or malignant process. The prevalence of AM is about 0.2 – 0.3% of all appendectomies (1). Although most cases are benign, they may bear risk of malignant conversion. However, the course of an appendiceal mucocele depends on its histologic characteristics. Five histologic subtypes have been defined: Mucosal hyperplasia; Simple or retention cysts; Mucinous adenomas (previously called cystadenomas); Mucinous appendiceal tumors of uncertain malignant potential (also known as LAMNs by the WHO); and Mucinous appendiceal adenocarcinoma (also known as cystadenocarcinoma). The first 3 subtypes run a benign course, whereas Mucinous appendiceal tumors of uncertain malignant show invasion into the muscularis mucosa and Mucinous appendiceal adenocarcinoma runs a malignant course (2). Therefore, accurate diagnosis and complete and precise resection is essential to prevent catastrophic complications. Concomitant occurrence of AM and ulcerative colitis (UC) is rare (2). The role of ulcerative colitis in occurrence of appendiceal mucocele is not known. Orta et al. reported an increased risk of AM in IBD patients who had colorectal cancer in their disease course. They suggested that A.M may be a presentation of colorectal cancer in these patients (3). Here, we present 3 cases of concomitant AM and UC. 

## Case Presentation

**Figure 1:**
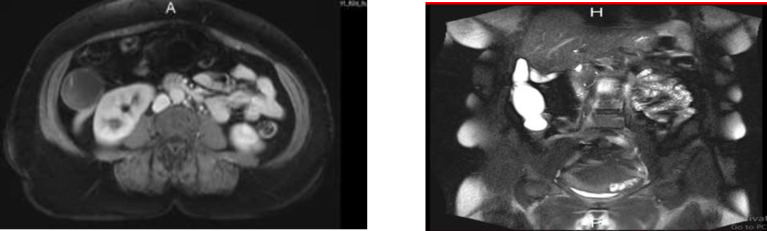
Abdomino-pelvic MRI demonstrating an elongated septated cystic-solid lesion at posterior aspect of cecum and ascending colon, and also adjacent to right liver lobe

**Case 1: **A 55-year-old woman with 2-year history of left sided UC referred for right lower quadrant (RLQ) pain lasting several months. She reported normal bowel movements and denied fever. Abdominal examination was not conclusive and abdominopelvic ultrasound showed an 11-cm elongated hypo-echoic mass in sub-hepatic area. Consequent colonoscopy was performed and reported left sided UC in remission (MAYO score 1) and the rest of the colon was normal apart from a prominence at the appendiceal orifice in her cecum. MRI showed an elongated 10 x 3 cm septated cystic-solid lesion at posterior aspect of cecum and ascending colon, and also adjacent to right liver lobe (figure 1). After surgical consultation, right hemicolectomy was done. Pathologic evaluation reported appendiceal mucocele of mucinous cyst adenoma type. No recurrence was reported during seven years of follow-up.

**Figure 2:**
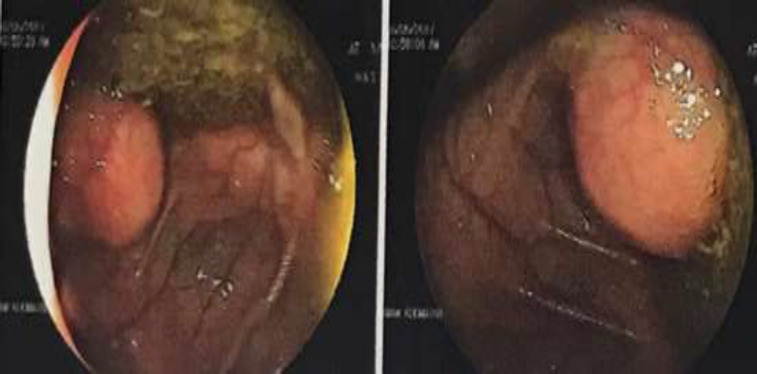
Colonoscopic evaluation demonstrating a glossy round protruding mass arising from appendiceal orifice

**Figure 3:**
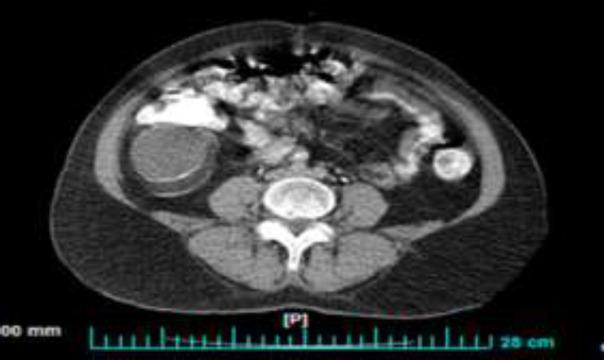
Abdomino-pelvic CT scan demonstrating a lobulated tubular calcified mass-like lesion with peripheral calcification in right lower quadrant

**Case 2: **A 52-year-old woman with 12-year history of pan-UC referred due to constant indolent RLQ pain during the previous few months. Abdominopelvic ultrasound assessment was normal. Colonoscopy showed moderate mucosal inflammation (MAYO score 4) in rectum, sigmoid and descending colon while transverse and ascending colon was normal. Similar to the former case, a 3 x 4-cm glossy round protruding mass arising from appendiceal orifice was seen in cecum (figure 2). Abdomino-pelvic CT scan reported a lobulated tubular calcified 57x54-mm mass-like lesion with peripheral calcification in RLQ (figure 3). After surgical consultation, right hemicolectomy was done. Pathologic evaluation showed a 14x8x3.5-cm low-grade mucinous neoplasm of appendix with intact serosa. Patient was followed for two years without any recurrence. 

**Case 3: **A 60-year-old man with 11-year history of UC pancolitis referred due to 5-month history of mild RLQ pain. Ultrasound suggested a 23-mm appendiceal mucocele. Abdomino-pelvic CT scan suggested a duplication cyst or a mesenteric cyst. Colonoscopy was performed and showed mucosal inflammation in the distal rectum and cecum. Other parts of colon and terminal ileum had normal mucosa and vascularity. Appendectomy was done and pathology showed cyst-adenoma type AM. There was no sign of recurrence during six-month follow-up.

## Discussion

Appendiceal mucocele is a rare disease and is only found in approximately 0.2 – 0.3% of all appendectomies (1). There are five different pathologic classes: 1) Retention cyst; 2) Mucous hyperplasia; 3) Mucinous Adenoma; 4) Mucinous appendiceal tumor of uncertain malignant potential [also known as low grade appendiceal mucinous neoplasm (LAMN) by WHO]; 5) Mucinous appendiceal adenocarcinoma (2). The first two types (retention cyst and mucous hyperplasia) do not lead to recurrence even if they are perforated during resection. However, those types that originate from neoplastic types (cyst adenoma, LAMN and cyst adenocarcinoma) secrete mucin and their perforation can lead to intraperitoneal spread of neoplastic calls and eventually pseudomyxoma peritonei (2). AM is more common among women and in those older than 50 years (2, 4). This characteristic was seen in our patients. Two out of our three patients were female and all were older than 50 years. 

The disease is sometimes asymptomatic, but it can present as a palpable mass, chronic RLQ pain or it can rarely present as altered bowel movements, weight loss, anemia, nausea, vomiting or even acute appendicitis (5). All our patients presented with chronic RLQ pain. In order to diagnose AM, the most important key is clinical suggestion. However, abdomino-pelvic ultrasound or CT scan can help in making the diagnosis by showing a cystic mass lesion in RLQ (6, 7). In our patients, both ultrasound and CT scan were helpful. Regarding treatment, all appendiceal mucoceles should be surgically resected, since even AMs with benign appearance may harbor carcinoma (8). Simple appendectomy is mostly 

Sufficient, but there are some criteria that indicate the need for hemi-colectomy instead of just simple appendectomy. These criteria are mostly used after pathologic evaluation of the lesion. They are: Involvement of the margins by the tumor; involvement of peri-appendiceal area or invasion to muscularis propria; metastasis to lymph nodes, tumor size > 2 cm; high-grade histology (poor differentiation or increased mitotic activity); involvement of appeandiceal base. Patients with any of the above-mentioned criteria are at high risk of recurrence and should undergo hemicooctomy (9, 10). Two of our patient underwent hemicolectomy due to large sizes of the lesions; and simple appendectomy was performed in only one patient. It should be mentioned that needle aspiration is not recommended since it can lead to seeding of neoplastic cells (2). Furthermore, laparoscopic excision of AM is not recommended (11) given that it may lead to peritoneal dissemination of appendiceal mucinous tumor (12). All our patients were also managed by laparotomy.

After surgical resection, neoplastic mucoceles (LAMN patients) should be followed annually by abdomino-pelvic CT scan and tumor markers including CEA, CA 19-9 and CA 125. These evaluations could be stopped after 5-10 years if no stigmata of recurrence or pseudomyxoma peritonei is found (13, 14). Our patients have been followed in regular visits and we have found no stigmata of recurrence yet. The role of ulcerative colitis in occurrence of appendiceal mucocele is not known. Orta et al. reported an increased risk of AM in IBD patients who had colorectal cancer in their disease course. They suggested that A.M may be a presentation of colorectal cancer in these patients (15). Also, Matsushita et al. reported higher prevalence of appendiceal cystadenoma in UC patients, compared with patients with crohn’s disease (16).

Furthermore, another explanation for the higher prevalence of AM in UC patients would be the obstruction of appendiceal orifice by inflammation of cecum, leading to formation of a mucocele (15, 17). Also, some suggest that even in patients with left-sided U.C, The orifice of appendix may have inflammation and this may justify the occurrence of AM in patients with left-sided U.C (17, 18). In our report, one of the patients had pancolitis with presence of inflammation in cecum and around appendiceal orifice at the time of diagnosis, but the other 2 patients had normal-looking mucosa of cecum. Therefore, we could not suggest the mechanism of appendiceal mucocele formation in these patients.

In conclusion, the mainstay of diagnosing appendiceal mucocele is proper clinical suspicion. Although concomitant occurrence of appendiceal mucocele and ulcerative colitis is rare, regarding the potential risk of malignancy in some subtypes of AM, the diagnosis is important for the decision on the surgical plan (simple appendectomy vs. colectomy). Furthermore, the correct diagnosis alerts the surgeon of the risks of rupture and avoids the development of pseudomyxoma peritonei. Therefore, gastroenterologists must keep in mind the diagnosis of appendiceal mucocele in UC patients with non-specific RLQ pain or those with palpable mass in RLQ; or in those patients with bulged or dilated appendiceal orifice during colonoscopy; or in the presence of a cystic lesion in RLQ through ultrasonography or CT-scan imaging. Our report adds new cases to the rarely reported cases of concomitant occurrence of UC and AM and emphasizes to the need for thorough evaluation of appendiceal orifice in UC patients.
